# Topical Application of Tea Tree Oil for the Treatment of Verruca Vulgaris

**DOI:** 10.2196/47107

**Published:** 2023-10-05

**Authors:** Deenadayalan Boopalan, Venugopal Vijayakumar, Poornima Ravi, Maheshkumar Kuppusamy

**Affiliations:** 1 Department of Neurology National Institute of Mental Health and Neurosciences Bengaluru India; 2 Department of Yoga Government Yoga and Naturopathy Medical College and Hospital Chennai India; 3 Department of Clinical Research Sri Ramachandra Institute of Higher Education and Research Chennai India; 4 Department of Physiology Government Yoga and Naturopathy Medical College and Hospital Chennai India

**Keywords:** aromatherapy, human papillomavirus, naturopathy, tea tree oil, warts, verruca vulgaris

## Introduction

Warts (verruca vulgaris) are benign epithelial proliferations associated with human papillomavirus (HPV) infection. They are a DNA virus belonging to the Papillomaviridae family [1]. Worldwide, 10% of the population is affected, and the prevalence is high in children attending school [2]. Common warts are excessive growths with an irregular surface ranging from 1 millimeter to several centimeters and are commonly seen on the upper and lower extremities [2]. Conventional treatment for warts includes topical application of salicylic acid, podophyllotoxin, trichloroacetic acid, formaldehyde, 5-fluorouracil, and photodynamic therapy [1]. Procedures such as cryotherapy, laser ablation, electrocautery, and surgical excision are painful and have higher chances of reoccurrence [1]. In aromatherapy, tea tree oil (TTO), extracted through steam distillation of the leaves and terminal branches of the Australian plant *Melaleuca alternifolia*, is used for the management of various dermatological conditions, including HPV [3,4], by naturopathy physicians. This letter aims to present TTO as a potential remedy for HPV warts, highlighting its properties, benefits, and the need for further research to establish its effectiveness and safety.

## Methods

We performed a comprehensive literature search to include original articles, case reports and case series, and abstracts that discussed the effect of TTO on HPV verruca vulgaris; in PubMed, Embase, and Google Scholar; and were published on April 9, 2022, or earlier. Reviews and articles that used TTO with other interventions were excluded. The following keywords were used: “tea tree oil” OR “aromatherapy” OR “naturopathy” AND “human papillomavirus” OR “HPV” OR “verruca vulgaris” OR “warts.”

## Results

A total of 4 articles involving 5 patients with warts treated with aromatherapy were included (Table 1). Warts were predominantly found on the upper and lower extremities, except for one case where the location was periungual [5]. The efficacy was assessed by using the visual analog scale [3] and clinical photographs daily [1,3]. Additionally, a follow-up was conducted to monitor for any recurrence [1]. All the studies included in this analysis reported complete clearance of warts.

**Table 1:** Characteristics of the studies included in the research letter.

Study, year, and participants		Country	Age (years)	Diagnosed by	Site of warts	Intervention details	Outcome
**Millar and Moore [4], 2008**							
	Female pediatric patient	United Kingdom	7	Dermatologist	Distal phalanges of the right middle finger	Topical application of tea tree oil once daily for 12 days	Complete clearance of warts without reoccurrence
**Alsanad and Alkhamees [3], 2016**		Saudi Arabia		Dermatologist			Complete clearance of warts without reoccurrence
	Male child		9		Left sole	Topical application of tea tree oil twice a day for 20 days	
	Male child		14		Proximal phalanges of the right little finger	Topical application of tea tree oil twice a day for 10 days	
**Lim et al [5], 2020**							
	Female child	South Korea	12	Dermatologist	Periungual and plantar	Topical application of tea tree oil for 9 months	Complete clearance of warts without reoccurrence
**Deenadayalan et al [1], 2022**							
	Adult female	India	22	Dermatologist	Distal phalanges of the right hand	Topical application of tea tree oil once a day for 7 days, afterward alternative day for 2 weeks	Complete clearance of warts without reoccurrence

## Discussion

This research letter indicates that TTO can be beneficial in treating warts, which are caused by HPV and commonly occur in pediatric and school-aged children. The potent anti-inflammatory and antiviral properties of TTO have been widely used to treat HPV infections. In vitro studies have also used TTO to treat herpes simplex virus [4]. Terpinene-4-ol and α-terpineol in TTO are known for their antiviral property and inhibit viral replication in both enveloped and nonenveloped viruses [4]. Terpinene-4-ol can inhibit the synthesis of proinflammatory cytokines, tumor necrosis factor, interleukin-1 (IL-1), IL-8, and prostaglandin E2 while increasing anti-inflammatory cytokines (IL-10 and IL-4), thereby reducing pain [1]. In addition to pain management and warts clearance, a pleasant scent may play a role in patient satisfaction. The treatment is also less expensive and has no adverse effects. The limitation of this letter was using single case reports with a small number of patients.

TTO should be considered a safe, cost-effective, noninvasive modality in the management of HPV warts. More research is necessary to understand the clinical applications and other long-term systemic effects.

## Abbreviations

HPV: human papillomavirus
IL: interleukin
TTO: tea tree oil

## References

[1] Deenadayalan B, Venugopal V, Maheshkumar K, Akila A, Priya CY (2022). Effect of topical application of tea tree oil (Melaleuca alternifolia) on hand warts. J Clin Diagn Res.

[2] Huang K, Li M, Xiao Y, Wu L, Li Y, Yang Y, Shi G, Yu N, Liu D, Su J, Wang X, Zhao S, Chen X (2022). The application of medical scale in the treatment of plantar warts: analysis and prospect. J Dermatolog Treat.

[3] Alsanad SM, Alkhamees OA (2016). Tea tree oil (Melaleuca alternifolia)-an efficient treatment for warts: two case reports. Int Arch Biomed Clin Res.

[4] Millar BC, Moore JE (2008). Successful topical treatment of hand warts in a paediatric patient with tea tree oil (Melaleuca alternifolia). Complement Ther Clin Pract.

[5] Lim HY, Yoon HJ, Ko WS (2020). A case of Verruca vulgaris in a paediatric patient treated with aroma therapy-based Korean medicine by tea tree oil (Melaleuca alternifolia). J Korean Med Ophthalmol Otolaryngol Dermatol.

